# Country-wide data of ecosystem structure from the third Dutch airborne laser scanning survey

**DOI:** 10.1016/j.dib.2022.108798

**Published:** 2022-12-05

**Authors:** W. Daniel Kissling, Yifang Shi, Zsófia Koma, Christiaan Meijer, Ou Ku, Francesco Nattino, Arie C. Seijmonsbergen, Meiert W. Grootes

**Affiliations:** a University of Amsterdam, Institute for Biodiversity and Ecosystem Dynamics (IBED), P.O. Box 94240, 1090 GE Amsterdam, The Netherlands; b LifeWatch ERIC, Virtual Laboratory and Innovations Centre (VLIC), University of Amsterdam Faculty of Science, Science Park 904, 1098 XH Amsterdam; c Aarhus University, Department of Biology, Center for Sustainable Landscapes Under Global Change, Ny Munkegade 116, 8000 Aarhus C, Denmark; d Netherlands eScience Center, Science Park 402 (Matrix III), 1098 XH Amsterdam, The Netherlands

**Keywords:** Ecosystem cover, Essential Biodiversity Variable, LiDAR metrics, Light detection and ranging, Point clouds, Structural complexity, Vegetation height, Vertical profile

## Abstract

The third Dutch national airborne laser scanning flight campaign (AHN3, Actueel Hoogtebestand Nederland) conducted between 2014 and 2019 during the leaf-off season (October–April) across the whole Netherlands provides a free and open-access, country-wide dataset with ∼700 billion points and a point density of ∼10(–20) points/m^2^. The AHN3 point cloud was obtained with Light Detection And Ranging (LiDAR) technology and contains for each point the x, y, z coordinates and additional characteristics (e.g. return number, intensity value, scan angle rank and GPS time). Moreover, the point cloud has been pre-processed by ‘Rijkswaterstraat’ (the executive agency of the Dutch Ministry of Infrastructure and Water Management), comes with a Digital Terrain Model (DTM) and a Digital Surface Model (DSM), and is delivered with a pre-classification of each point into one of six classes (0: Never Classified, 1: Unclassified, 2: Ground, 6: Building, 9: Water, 26: Reserved [bridges etc.]). However, no detailed information on vegetation structure is available from the AHN3 point cloud. We processed the AHN3 point cloud (∼16 TB uncompressed data volume) into 10 m resolution raster layers of ecosystem structure at a national extent, using a novel high-throughput workflow called ‘Laserfarm’ and a cluster of virtual machines with fast central processing units, high memory nodes and associated big data storage for managing the large amount of files. The raster layers (available as GeoTIFF files) capture 25 LiDAR metrics of vegetation structure, including ecosystem height (e.g. 95^th^ percentiles of normalized z), ecosystem cover (e.g. pulse penetration ratio, canopy cover, and density of vegetation points within defined height layers), and ecosystem structural complexity (e.g. skewness and variability of vertical vegetation point distribution). The raster layers make use of the Dutch projected coordinate system (EPSG:28992 Amersfoort / RD New), are each ∼1 GB in size, and can be readily used by ecologists in a geographic information system (GIS) or analytical open-source software such as R and Python. Even though the class ‘1: Unclassified’ mainly includes vegetation points, other objects such as cars, fences, and boats can also be present in this class, introducing potential biases in the derived data products. We therefore validated the raster layers of ecosystem structure using >180,000 hand-labelled LiDAR points in 100 randomly selected sample plots (10 m × 10 m each) across the Netherlands. Besides vegetation, objects such as boats, fences, and cars were identified in the sampled plots. However, the misclassification rate of vegetation points (i.e. non-vegetation points that were assumed to be vegetation) was low (∼0.05) and the accuracy of the 25 LiDAR metrics derived from the AHN3 point cloud was high (∼90%). To minimize existing inaccuracies in this country-wide data product (e.g. ships on water bodies, chimneys on roofs, or cars on roads that might be incorrectly used as vegetation points), we provide an additional mask that captures water bodies, buildings and roads generated from the Dutch cadaster dataset. This newly generated country-wide ecosystem structure data product provides new opportunities for ecology and biodiversity science, e.g. for mapping the 3D vegetation structure of a variety of ecosystems or for modelling biodiversity, species distributions, abundance and ecological niches of animals and their habitats.

**Specifications Table**

**Table:** 

Subject	Environmental Science, Ecology
Specific subject area	Macroecology and geographical ecology: geospatial information on the 3D structure of ecosystems is essential for modelling the broad-scale distribution of life on Earth.
Type of data	Image (GeoTIFF files in the Dutch projected coordinate system (EPSG:28992 Amersfoort / RD New)
How the data were acquired	We acquired the raw LiDAR data (AHN3 point cloud dataset) from the repository of the PDOK webservices (https://app.pdok.nl/ahn3-downloadpage/) using a script for automatic downloading (available on GitHub: https://github.com/eEcoLiDAR/downloadAHN). We subsequently processed the multi-terabyte point clouds from AHN3 with the ‘Laserfarm’ workflow (https://pypi.org/project/laserfarm/) into 25 raster layers of ecosystem structure at a national extent with 10 m spatial resolution. In brief, the Laserfarm workflow (1) splits the raw data into tiles of appropriate size based on a defined grid (re-tiling), (2) calculates the normalized vegetation height for each individual point as the height relative to the lowest point within a defined grid cell (normalization), (3) calculates 25 LiDAR metrics with a defined spatial resolution (feature extraction), and (4) merges all tiles for each metric into raster layers in GeoTIFF format (rasterization). A detailed description of this high-throughput LiDAR workflow is provided in a paper describing the Laserfarm design, implementation and its performance [1]. In addition to the 25 LiDAR metrics, we calculated the AHN3 point density (# of points) for each 10 m x 10 m grid cell using the point density feature from the ‘Laserchicken’ software [2] which is also incorporated into the Laserfarm workflow [1]. Finally, we derived a mask (with 10 m spatial resolution) capturing water surfaces and human infrastructures (e.g. buildings and roads) by aggregating and rasterizing the water, building and road polygons from the shapefiles of the 2018 Dutch cadaster data (TOP10NL, https://zakelijk.kadaster.nl/-/top10nl). For this step, a Jupyter Notebook was employed which is available on GitHub (https://github.com/eEcoLiDAR/AHN/tree/main/AHN-mask).
Data format	Raster layers in GeoTIFF format (10 m spatial resolution) derived from (1) processing raw data (AHN3 point clouds) into LiDAR metrics capturing different aspects of ecosystem structure (25 raster layers), (2) calculating AHN3 point density (1 raster layer), and (3) aggregating water surfaces and human infrastructures (e.g. buildings and roads) from shapefiles of the Dutch TOP10NL cadaster data (1 raster layer).
Description of data collection	Only the AHN3 class ‘1: Unclassified’ includes vegetation points. Normalizing the height (z-values) of all points in the AHN3 class ‘1: Unclassified’ relative to the terrain surface was done with the lowest point within a 1 m × 1 m cell using the ‘Normalize’ module of 'Laserchicken' (https://laserchicken.readthedocs.io/en/latest/#normalize).
Data source location	The raw data (LiDAR point clouds) have been obtained by the third Dutch national airborne laser scanning flight campaign (AHN3). They can be viewed either via the Dutch geodataset platform called ‘Publieke Dienstverlening Op de Kaart (PDOK)’ (https://www.pdok.nl/introductie/-/article/actueel-hoogtebestand-nederland-ahn3-) or via the viewer of the ‘Actueel Hoogtebestand Nederland (AHN)’ (https://ahn.arcgisonline.nl/ahnviewer/). Besides the raw LiDAR point clouds, a Digital Terrain Model (DTM) and a Digital Surface Model (DSM) at both 0.5 m and 5 m resolution are also provided by the Actueel Hoogtebestand Nederland (AHN). This information is publicly available (https://app.pdok.nl/ahn3-downloadpage/), and we therefore do not provide any topographic data with our data publication. Additional raw data (polygon shapefiles) for creating the mask of water surfaces and human infrastructures (buildings and roads) were obtained from the 2018 Dutch cadastre data (TOP10NL), also available from PDOK (https://www.pdok.nl/introductie/-/article/basisregistratie-topografie-brt-topnl).
Data accessibility	All data (i.e. 27 raster layers in GeoTIFF format, see section 'Data description' below) are made publicly available [3]. Repository name: Zenodo Data identification number: DOI 10.5281/zenodo.6421381 Direct URL to data: https://zenodo.org/record/6421381
Related research article	Kissling, W.D., Shi, Y., Koma, Z., Meijer, C., Ku, O., Nattino, F., Seijmonsbergen, A.C., Grootes, M.W., 2022. Laserfarm – A high-throughput workflow for generating geospatial data products of ecosystem structure from airborne laser scanning point clouds. *Ecological Informatics* 72, 101836. https://doi.org/10.1016/j.ecoinf.2022.101836

**Value of the Data**

• Ecosystem structure data are important for understanding, modelling and predicting biodiversity because the species richness, composition, distribution and abundance of organisms and their habitat preferences (e.g. nest sites and shelter), food provisioning and foraging are tightly linked to the horizontal and vertical heterogeneity of vegetation.
• The physical structure of ecosystems also influences microclimates at the land–air interface via respiration, heat and energy exchange. This affects species behavior, growth, reproduction, and survival. Predictions of species and ecosystem responses to global change thus require high resolution data of ecosystem structure to account for temperature buffering near the ground and microrefugia within landscapes.
• Measurements of the vertical structure of forests and other ecosystems are also critical for accurately assessing biomass and carbon storage, and how land use changes, ecosystem restoration or variations in climate may impact atmospheric CO_2_ concentrations.
• Spatially contiguous, high resolution raster layers of ecosystem structure will thus be beneficial for a range of users, including field biologists, ecologists, conservationists, ecological modelers, geoinformaticians, land managers and environmental system analysts. Researchers from other domains (e.g. hydrology and climatology) might also take advantage of such data.
• Ecosystem structure data can be used as predictors in statistical models (e.g. profile models, regressions, machine learning and geographical models) to correlate species observations with environmental layers. Such predictive habitat distribution models aim to quantify and map the determinants of species’ ecological niches and their ability to cope with climate or land use change.

## 1 Objective

This dataset was generated during the development of the Laserfarm workflow, a high-throughput pipeline for generating geospatial data products of ecosystem structure from airborne laser scanning point clouds. The aim was to create a high-resolution (10 m) geospatial dataset of 25 LiDAR metrics (i.e. raster layers in GeoTIFF format) from multi-terabyte LiDAR point clouds at a national extent (i.e. across the Netherlands), capturing various aspects of ecosystem structure, including vegetation height, cover and structural complexity of vegetation. This provides a standardized set of Essential Biodiversity Variables (EBVs) derived from LiDAR for the EBV ‘Ecosystem Vertical Profile’ [4,5] and thereby facilitates the monitoring and modelling of biodiversity and ecosystems [6], [7], [8]. The original research article related to this data publication describes the design principles, architecture, implementation and performance of the Laserfarm workflow, and the statistical relationships among the 25 LiDAR metrics. Here, we describe the specific details and mathematical description of each LiDAR metric, perform a validation with hand-labelled points to quantify the misclassification rate and the accuracy of the LiDAR metrics, and provide a mask of water surfaces and human infrastructures (buildings and roads) from the Dutch cadaster data to minimize inaccuracies related to misclassifications. The overall objective of this data paper is therefore to provide an open-access dataset of ecosystem structure variables for modeling species distributions [9,10] and ecological niches [11], for analyzing biodiversity in relation to vegetation structure and land use [12], and for mapping land cover types [13] and other habitat features such as hedges and tree lines [14].

## 2 Data Description

### 2.1 Raster layers of ecosystem structure

A total of 25 LiDAR metrics of ecosystem structure were calculated (Table 1). The spatial resolution of grid cells was 10 m and the spatial extent was the whole Netherlands. The AHN3 point cloud was used as input (see above 'Data source location') and metric calculation was performed with the Laserfarm workflow (https://pypi.org/project/laserfarm/), using the feature extraction module from the 'Laserchicken' software (https://laserchicken.readthedocs.io/en/latest/#features). For each of the 25 calculated LiDAR metrics, the GeoTIFF file name, the Laserchicken feature name, the formula, a general description and its ecological relevance is provided (Table 1). The metrics are grouped into three key dimensions of ecosystem structure, following a standardized framework of ecosystem structure variables in the context of EBVs [4], namely ecosystem height, ecosystem cover and ecosystem structural complexity. All metrics were calculated with the normalized point cloud using predominantly vegetation points (class ‘unclassified’ from AHN3 classification provided by ‘Rijkswaterstraat’), except the pulse penetration ratio which additionally requires ground points.

**Table 1:** Twenty-five LiDAR metrics capturing ecosystem structure in three key dimensions (ecosystem height, ecosystem cover and ecosystem structural complexity). All metrics were calculated with the normalized point cloud, using the Dutch AHN3 point clouds as input and the features from the 'Laserchicken' software (https://laserchicken.readthedocs.io/en/latest/#features). More details on metric calculation are provided on GitHub (https://github.com/eEcoLiDAR/laserchicken) and on the 'Laserchicken' documentation page (https://laserchicken.readthedocs.io/en/latest/). The processed 10 m resolution GeoTIFF files are available from the Zenodo repository [3].

LiDAR metric (abbreviation)	GeoTIFF file name in Zenodo	Laserchicken feature name	Formula	Description	Ecological relevance
*Ecosystem height*					
Maximum vegetation height (Hmax)	ahn3_10m_max_ normalized_height	max_norm_z	$z_{max}$	Maximum of normalized z within a grid cell	Height of the vegetation canopy surface and tree tops
Mean of vegetation height (Hmean)	ahn3_10m_mean_ normalized_height	mean_norm_z	$\frac{1}{N}\times \sum z_{i}$ where N is the number of normalized z values and $\sum z_{i}$ the sum of all normalized z values in a grid cell	Mean of normalized z within a grid cell	Average height of vegetation (e.g. mean tree and shrub height in forests)
Median of vegetation height (Hmedian)	ahn3_10m_median_ normalized_height	median_norm_z	$z_{median}$	Median of normalized z within a grid cell	Average height and vertical distribution of vegetation
25^th^ percentile of vegetation height (Hp25)	ahn3_10m_perc_25_ normalized_height	perc_25_normalized_ height	$n\;=(\frac{25}{100})\times N$, where N = number of normalized z values (sorted from smallest to largest), and n = ordinal rank of a given value	Capturing the 25^th^ percentile of normalized z within a grid cell	Density of vegetation in the low stratum
50^th^ percentile of vegetation height (Hp50)	ahn3_10m_perc_50_ normalized_height	perc_50_normalized_ height	$n\;=(\frac{50}{100})\times N$, where N = number of normalized z values (sorted from smallest to largest), and n = ordinal rank of a given value. This corresponds to the Hmedian	Capturing the 50^th^ percentile of normalized z within a grid cell	Average height and vertical distribution of vegetation
75^th^ percentile of vegetation height (Hp75)	ahn3_10m_perc_75_ normalized_height	perc_75_normalized_ height	$n\;=(\frac{75}{100})\times N$, where N = number of normalized z values (sorted from smallest to largest), and n = ordinal rank of a given value	Capturing the 75^th^ percentile of normalized z within a grid cell	Density of vegetation in the upper stratum
95^th^ percentile of vegetation height (Hp95)	ahn3_10m_perc_95_ normalized_height	perc_95_normalized_ height	$n\;=(\frac{95}{100})\times N$, where N = number of normalized z values (sorted from smallest to largest), and n = ordinal rank of a given value	Capturing the 95^th^ percentile of normalized z within a grid cell	Height of the vegetation canopy surface and tree tops, accounting for the effect of outliers
*Ecosystem cover*					
Pulse penetration ratio (PPR)	ahn3_10m_pulse_ penetration_ratio	pulse_penetration_ ratio	$\frac{N_{ground}}{N_{total}}$	Ratio of number of ground points ($N_{ground}$) to the total number of points ($N_{total}$) within a grid cell	Openness of vegetation, canopy fractional cover, laser penetration index
Canopy cover above mean height (Density_above_mean_z)	ahn3_10m_density_absolute_ mean_ normalized_height	density_absolute_ mean_norm_z	$100\;\times \;\sum [z_{i}>\bar{z}]/N$ where $z_{i}$ are all normalized z values that are larger than the mean vegetation height $\bar{z}$ within a grid cell, and N the total number of normalized z values	Number of returns above mean height within a grid cell	Density of upper vegetation layer
Density of vegetation points below 1 m (BR_below_1)	ahn3_10m_band_ratio_ normalized_height_1	band_ratio_normalized_ height<1	$N_{z<1}/N_{total}$	Ratio of number of vegetation points (<1 m) to the total number of vegetation points within a grid cell	Density of vegetation <1 m
Density of vegetation points between 1–2 m (BR_1_2)	ahn3_10m_band_ratio_1_ normalized_height_2	band_ratio_1<normalized_ height<2	$N_{1<z<2}/N_{total}$	Ratio of number of vegetation points (between 1–2 m) to the total number of vegetation points within a grid cell	Density of vegetation in 1–2 m layer
Density of vegetation points between 2–3 m (BR_2_3)	ahn3_10m_band_ratio_2_ normalized_height_3	band_ratio_2<normalized_ height<3	$N_{2<z<3}/N_{total}$	Ratio of number of vegetation points (between 2–3 m) to the total number of vegetation points within a grid cell	Density of vegetation in 2–3 m layer
Density of vegetation points above 3 m (BR_above_3)	ahn3_10m_band_ratio_3_ normalized_height	band_ratio_normalized_ height>3	$N_{z>3}/N_{total}$	Ratio of number of vegetation points (>3 m) to the total number of vegetation points within a grid cell	Density of vegetation above 3 m
Density of vegetation points between 3–4 m (BR_3_4)	ahn3_10m_band_ratio_3_ normalized_height_4	band_ratio_3<normalized_ height<4	$N_{3<z<4}/N_{total}$	Ratio of number of vegetation points (between 3–4 m) to the total number of vegetation points within a grid cell	Density of vegetation in 3–4 m layer
Density of vegetation points between 4–5 m (BR_4_5)	ahn3_10m_band_ratio_4_ normalized_height_5	band_ratio_4<normalized_ height<5	$N_{4<z<5}/N_{total}$	Ratio of number of vegetation points (between 4–5 m) to the total number of vegetation points within a grid cell	Density of vegetation in 4–5 m layer
Density of vegetation points below 5 m (BR_below_5)	ahn3_10m_band_ratio_ normalized_height_5	band_ratio_normalized_ height<5	$N_{z<5}/N_{total}$	Ratio of number of vegetation points (<5 m) to the total number of vegetation points within a grid cell	Density of vegetation in understory layer (<5 m)
Density of vegetation points between 5–20 m (BR_5_20)	ahn3_10m_band_ratio_5_ normalized_height_20	band_ratio_5<normalized_ height<20	$N_{5<z<20}/N_{total}$	Ratio of number of vegetation points (between 5–20 m) to the total number of vegetation points within a grid cell	Density of vegetation in 5–20 m layer
Density of vegetation points above 20 m (BR_above_20)	ahn3_10m_band_ratio_20_ normalized_height	band_ratio_normalized_ height>20	$N_{z>20}/N_{total}$	Ratio of number of vegetation points (>20 m) to the total number of vegetation points within a grid cell	Density of vegetation above 20 m
*Ecosystem structural complexity*					
Coefficient of variation of vegetation height (Coeff_var_z)	ahn3_10m_coeff_var_ normalized_height	coeff_var_norm_z	$\frac{1}{\bar{Z}}\times \;\sqrt{\sum \frac{{\left(z_{i}-\bar{z}\right)}^{2}}{N-1}}$ where $\bar{z}$ is the mean vegetation height, $z_{i}$ all normalized z values in a grid cell, and N the number of normalized z values	Coefficient of variation of normalized z within a grid cell	Vertical variability of vegetation distribution (ratio of the standard deviation to the mean)
Shannon index (Entropy_z)	ahn3_10m_entropy_ normalized_height	entropy_norm_z	$-\sum_{i}p_{i}\times log_{2}p_{i}$ where $p_{i}=N_{i}/\sum_{j}N_{j}$ and $N_{i}$ the points in bin i	The negative sum of the proportion of points within 0.5 m height layers multiplied with the logarithm of the proportion of points within 0.5 m height layers within a grid cell	Complexity and evenness of vertical vegetation distribution, sometimes referred to as foliage height diversity
Kurtosis of vegetation height (Hkurt)	ahn3_10m_kurto_ normalized_height	kurto_norm_z	$\frac{1}{\sigma^{4}}\times \sum {\left(z_{i}-\bar{z}\right)}^{4}/N\;$where $z_{i}$ are the normalized z values in a grid cell, $\bar{z}$ the mean of normalized z values, and N the total number of normalized z values	Kurtosis of normalized z within a grid cell	Vertical distribution (‘tailedness’) of vegetation
Roughness of vegetation (Sigma_z)	ahn3_10m_sigma_z	sigma_z	$\sqrt{\sum {\left(R_{i}-\bar{R}\right)}^{2}/\left(N-1\right)}$ where $R_{i}$ are the residual after plane fitting, and $\bar{R}$ the mean of residuals	Standard deviation of the residuals of a locally fitted plane within a cylinder	Small-scale roughness and variability of vegetation
Skewness of vegetation height (Hskew)	ahn3_10m_skew_ normalized_height	skew_norm_z	$\frac{1}{\sigma^{3}}\times \sum {\left(z_{i}-\bar{z}\right)}^{3}/N\;$where $z_{i}$ are the normalized z values in a grid cell, $\bar{z}$ the mean of normalized z values, and N the total number of normalized z values	Skewness of normalized z within a grid cell	Vertical distribution (asymmetry) of vegetation
Standard deviation of vegetation height (Hstd)	ahn3_10m_std_ normalized_height	std_norm_z	$\sqrt{\sum \frac{{\left(z_{i}-\bar{z}\right)}^{2}}{N-1}}$ where $\bar{z}$ is the mean vegetation height, $z_{i}$ all normalized z values in a grid cell, and N the number of normalized z values	Standard deviation of normalized z within a grid cell	Vertical variability (i.e. amount of variation around mean) of vegetation distribution
Variance of vegetation height (Hvar)	ahn3_10m_var_ normalized_height	var_norm_z	$\sum \frac{{\left(z_{i}-\bar{z}\right)}^{2}}{N-1}$ where $\bar{z}$ is the mean vegetation height, $z_{i}$ all normalized z values in a grid cell, and N the number of normalized z values	Variance of normalized z within a grid cell	Vertical variability of vegetation distribution (dispersion around mean height)

### 2.2 Validation

For generating the 25 LiDAR metrics of ecosystem structure we used the ASPRS classification code ‘1: Unclassified’ [15] of the AHN3 point cloud to represent vegetation points. This may introduce biases into the generated data products if the class contains not only vegetation points but also other objects such as cars, fences, poles, boats, etc. To validate the derived LiDAR metrics of ecosystem structure we randomly selected 100 sample plots (10 m × 10 m each) across the Netherlands and hand-labelled the segmented point clouds (183,837 points in total) into three classes: vegetation, ground, and others (e.g. buildings, cars, fences). We then used these hand-labelled points as ground truth and calculated two validation metrics: (1) the misclassification rate for each plot (i.e. the number of points incorrectly classified as vegetation / total number of points in the ASPRS class ‘1: Unclassified’), and (2) the accuracy of the 25 derived LiDAR metrics (i.e. the number of plots in which the LiDAR metric calculation did not differ between hand-labelled and originally classified points / total number of plots).

**Fig. 1:**
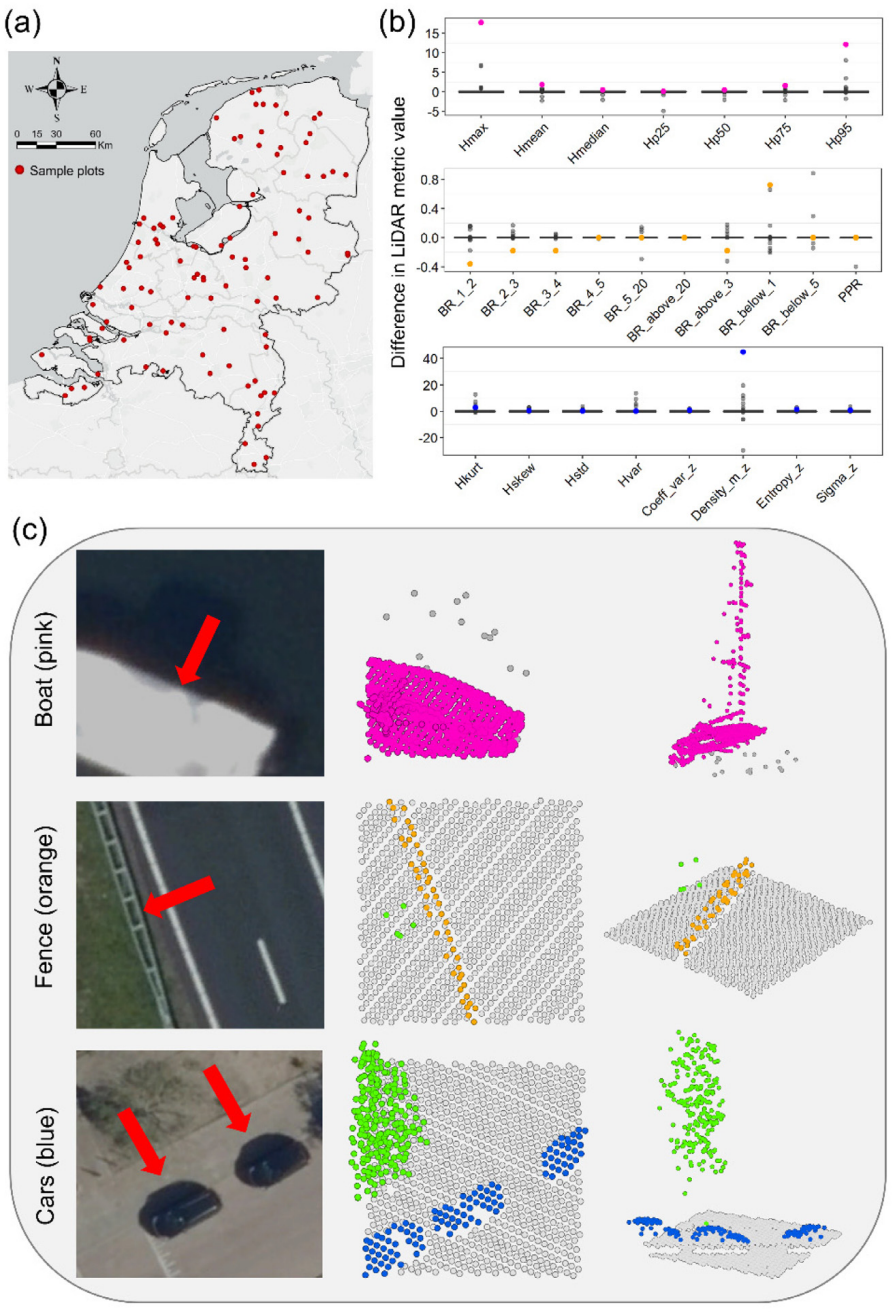
Accuracy assessment of 25 LiDAR metrics of ecosystem structure. (a) Locations of 100 randomly selected 10 m × 10 m plots in the Netherlands. (b) Boxplots showing the differences in LiDAR metric values between calculations using the hand-labelled vegetation points and calculations using the points from the original ASPRS classification (class ‘1: Unclassified’). The units of the y-axes correspond to the units of each individual metric (e.g. meter for Hmax) and are 0 if LiDAR metric calculations of the hand-labelled points and the original classification are the same. (c) Examples of 10 m × 10 m plots showing points belonging to boats (pink), road fences (orange), and cars (blue). These points belong to the ASPRS classification (class ‘1: Unclassified’) and can bias LiDAR metrics of ecosystem structure if this class is assumed to contain only vegetation. Green points are examples of true vegetation points. Colors correspond to panel (b) in which the inaccuracies in LiDAR metrics resulting from incorrectly classified vegetation points in these plots are shown.

The 100 randomly selected 10 m × 10 m plots were widely spread across the whole Netherlands (Fig. 1a). Most plots (88%) did not contain any misclassification (i.e. exclusively true vegetation points within the ASPRS class ‘1: Unclassified’). Four plots (4%) had more than half of the points in the ASPRS class ‘1: Unclassified’ incorrectly classified as vegetation. Across all 100 plots the misclassification rate was very low (0.05 ± 0.19, *n* = 100).

As a consequence of the low misclassification rate, only a few plots showed differences in the LiDAR metric calculation between the hand-labelled vegetation points and the points originally classified as ‘1: Unclassified’ (see dots in Fig. 1b). Overall, the accuracy of the generated LiDAR metrics was high (0.90 ± 0.04, *n* = 25 LiDAR metrics), ranging from 0.87–1. The number of plots with differences in LiDAR metric values and the degree of difference varied among LiDAR metrics (Fig. 1b). For instance, the LiDAR metrics Hmax and Hp95 showed the strongest differences among height-related LiDAR metrics, whereas BR_below_1 and BR_below_5 showed the strongest differences among metrics characterizing the density of vegetation points in certain vegetation layers (Fig. 1b). Closer inspection of specific plots with misclassifications showed that incorrectly classified points mainly belonged to boats, fences, and cars (Fig. 1c).

### 2.3 Point density

Besides the 25 LiDAR metrics, we additionally calculated the point density for each 10 m x 10 m grid cell to quantify the variability in available AHN3 point densities across the Netherlands. This represents the spatial distribution of the point cloud density and can be used for additional analyses, e.g. to test how LiDAR metrics of ecosystem structure vary with point densities. The AHN3 point density was calculated for each 10 m x 10 m grid cell using the ‘point_density’ feature from the 'Laserchicken' software (https://laserchicken.readthedocs.io/en/latest/#features).

Formal description: N/A where N is the total number of points (including points from all classes from the classification, i.e. not only the points from the class ‘unclassified’) and A is the area (here: 10 m x 10 m grid cell).

### 2.4 Mask

**Fig. 2:**
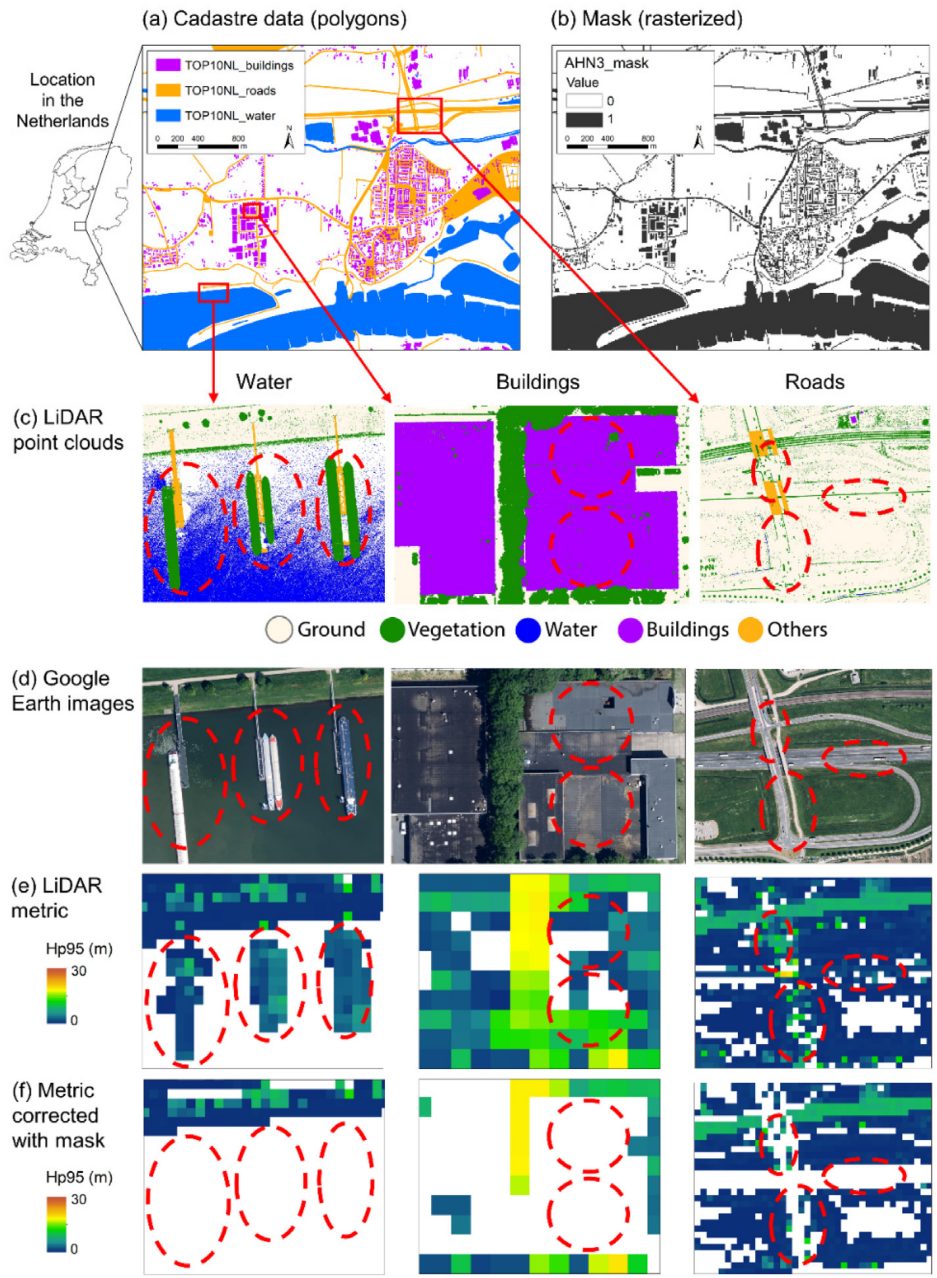
Examples of minimizing errors in calculated LiDAR metrics using a mask derived from rasterized cadastre data. (a) Shapefiles of water, building and road polygons from the 2018 Dutch cadaster data (TOP10NL). (b) Mask generated from rasterizing the cadaster data. (c) LiDAR point clouds from the third Dutch airborne laser scanning survey (AHN3) illustrating example areas with water, buildings and roads. The classification code that includes vegetation points (green) can also contain other objects such as ships on water, chimneys on building roofs, and cars on roads (examples highlighted with red stippled circles). (d) Google Earth images from the example areas. Note that the date of these images does not correspond exactly with the date of the airborne laser scanning survey. (e) LiDAR metric of ecosystem height (Hp95 = 95^th^ percentile of normalized *z*) extracted and rasterized from the point cloud. (f) Same LiDAR metric corrected with mask. Red stippled circles illustrate areas where misrepresentation of vegetation (on water, roofs, and roads, respectively) has been corrected.

We additionally derived a mask of water surfaces and human infrastructures (buildings and roads) from the Dutch cadaster data which allows the user to minimize inaccuracies related to misclassifications, e.g. ships on water surface, chimneys on roofs, or cars on roads which might incorrectly be considered as vegetation. A mask of water surfaces and human infrastructures (buildings and roads) was created from the shapefiles of the 2018 Dutch cadaster data (TOP10NL, https://zakelijk.kadaster.nl/-/top10nl) using the same spatial resolution (i.e. 10 meter) and projected coordinate system (EPSG:28992 Amersfoort / RD New) as the other GeoTIFF files. We aggregated the water, building and road polygons from the TOP10NL shapefiles into one type, and rasterized them using a binary classification (1: water, building and roads; 0: other). The mask allows users to minimize errors in the generated country-wide ecosystem structure data because not all points in the ASPRS point class ‘1: Unclassified’ are vegetation points (Fig. 2).

## 3 Experimental Design, Materials and Methods

### 3.1 Raw data

The whole LiDAR point cloud dataset from AHN3 is large and contains ∼700 billion points and ∼16 TB uncompressed data volume, available in 1,367 point cloud files (LAZ format). We downloaded all 1,367 files from the AHN3 repository using the PDOK webservices (https://app.pdok.nl/ahn3-downloadpage/) and a script for automatic download (https://github.com/eEcoLiDAR/downloadAHN). Data were downloaded to the GRID storage infrastructure (http://doc.grid.surfsara.nl/en/latest/Pages/Advanced/grid_storage.html) from SURF, the trans-national IT infrastructure for the Dutch academic community (https://www.surf.nl/en/ict-facilities).

### 3.2 Processing

For processing the files, we set-up a cluster of 11 virtual machines (VMs) using the HPC Cloud from SURF (https://userinfo.surfsara.nl/systems/hpc-cloud). Each of the 11 VMs had 2 cores (thus a total of 22 cores), 32 GB or 64 GB RAM, and 256 GB local HDD. We used the Laserfarm workflow (version 0.1.5) available from PyPI (https://pypi.org/project/laserfarm/), GitHub (https://github.com/eEcoLiDAR/Laserfarm) and Zenodo (https://zenodo.org/record/5636773) to process the multi-terabytes of AHN3 point clouds into GeoTIFF files of LiDAR metrics capturing ecosystems structure. The Laserfarm workflow is implemented in Python and makes use of open-source tools such as ‘Laserchicken’ [2], the Point Data Abstraction Library (PDAL, https://pdal.io/), the Geospatial Data Abstraction Library (GDAL, https://gdal.org/), the Dask library [16], and numerous packages hosted on the open source Python Package Index (PyPI, https://pypi.org/). All steps of the AHN3 processing using the Laserfarm workflow are available in Jupyter Notebooks (https://github.com/eEcoLiDAR/AHN/tree/main/AHN3) and involved the following processing steps:

As a first step (‘Re-tiling’), we split the 1,367 LAZ files into a grid with 1 km × 1 km size (512 × 512 cells across the Netherlands), making use of the Dutch projected coordinate system (EPSG:28992 Amersfoort / RD New). The LAZ files were retrieved from the storage and then split using functionality from the PDAL library [17]. This resulted in 37,457 tiles for further processing.

In the second step (‘Normalization’), we calculated the normalized height for each individual point as the height relative to the lowest point within a 1 m × 1 m cell. This pipeline employs the ‘Normalize’ module of the 'Laserchicken' software [2].

In the third step (‘Feature extraction’), we calculated the LiDAR metrics using the ‘Features’ and 'Compute Neighbors' modules of the 'Laserchicken' software [2]. We focused on 25 LiDAR metrics capturing ecosystem height, ecosystem cover and ecosystem structural complexity (see details above in 'Data description'). We used the ASPRS classification code ‘1: Unclassified’ [15] of the AHN3 point cloud to represent vegetation points. We defined the spatial resolution (grid cell size) for all metrics as 10 m × 10 m using the centroids of square cells and an infinite vertical extent as the volume geometry in the 'Laserchicken' software [2]. We finally generated 37,457 PLY files for each LiDAR metric and exported them to separate folders.

In the fourth step (‘Rasterization’), we merged and exported the PLY tiles for each metric as a single-band GeoTIFF file in the Dutch projected coordinate system (EPSG:28992 Amersfoort / RD New).

### 3.3 Validation

We randomly located 100 plots (each of 10 m × 10 m size) across the Netherlands using the sampleRandom() function in R (https://www.rdocumentation.org/packages/raster/versions/3.5-15/topics/sampleRandom). We then segmented the point cloud of each plot from the raw AHN3 point clouds using the ‘lasclip’ tool from the Lastools software (https://rapidlasso.com/lastools/), using the polygons of the sampled plots. We then hand-labelled all segmented point clouds (i.e. 183,837 points in total) into the classes of vegetation, ground, and others (e.g. buildings, cars, fences). This was done with the ArcGIS Pro interactive editing tool for LAS classification (see https://pro.arcgis.com/en/pro-app/latest/help/data/las-dataset/interactive-las-class-code-editing.htm). Each of the 25 LiDAR metrics was then calculated for each plot using the Laserfarm workflow, once using all points from the ASPRS point class ‘1: Unclassified’ and once using only the vegetation points from the hand-labelled point clouds (as ground truth). The values of each LiDAR metric (in GeoTIFF layers from both the original point clouds and the hand-labelled point clouds) for each plot were then extracted using the extract() function in R (https://www.rdocumentation.org/packages/raster/versions/3.5-15/topics/extract).

The misclassification rate was calculated for each plot as the number of points which were incorrectly classified as vegetation divided by the total number of points in the ASPRS class ‘1: Unclassified’. Accuracy of the 25 LiDAR metrics was assessed by taking the number of plots in which the LiDAR metric calculation did not differ between hand-labelled and originally classified points (i.e. difference = 0) and dividing it by the total number of plots (*n* = 100). This allowed us to quantify how accurately the 25 LiDAR metrics capture vegetation structure, and to what extend their values might be affected by non-vegetation points that remain in the class ‘unclassified’ of the AHN3 pre-classification.

### 3.4 Mask

The mask layer was generated using the water, buildings and road polygons from the TOP10NL cadaster data (see above ‘Data source location’). We aggregated the polygons and exported them as a raster layer with a binary classification (1: water, building and roads; 0: other) and 10 m resolution, using a Jupyter Notebook (https://github.com/eEcoLiDAR/AHN/tree/main/AHN-mask).

## Ethics Statements

This work meets the requirements for ethical publishing (https://www.elsevier.com/authors/policies-and-guidelines). The work does not include chemicals, procedures or equipment that have any unusual hazards inherent in their use, nor does it involve the use of animal or human subjects. No studies on patients or volunteers have been performed.

## CRediT authorship contribution statement

**W. Daniel Kissling:** Conceptualization, Formal analysis, Funding acquisition, Project administration, Supervision, Visualization, Writing – original draft, Writing – review & editing. **Yifang Shi:** Data curation, Formal analysis, Methodology, Validation, Visualization, Writing – review & editing. **Zsófia Koma:** Investigation, Methodology, Writing – review & editing. **Christiaan Meijer:** Software, Validation, Methodology. **Ou Ku:** Software, Validation, Methodology. **Francesco Nattino:** Software, Validation, Formal analysis, Methodology. **Arie C. Seijmonsbergen:** Funding acquisition, Writing – review & editing. **Meiert W. Grootes:** Conceptualization, Methodology, Resources, Software, Supervision, Validation, Writing – review & editing.

## Declaration of Competing Interest

The authors declare that they have no known competing financial interests or personal relationships that could have appeared to influence the work reported in this paper.

## Data Availability

Country-wide data products for the ecosystem structure metrics derived from ALS data across the Netherlands (AHN3) (Original data) (ZENODO)
